# Adjudication of a primary trial outcome: Results of a calibration exercise and protocol for a large international trial

**DOI:** 10.1016/j.conctc.2024.101284

**Published:** 2024-03-05

**Authors:** Deborah Cook, Adam Deane, Joanna C. Dionne, François Lauzier, John C. Marshall, Yaseen M. Arabi, M. Elizabeth Wilcox, Marlies Ostermann, Abdulrahman Al-Fares, Diane Heels-Ansdell, Nicole Zytaruk, Lehana Thabane, Simon Finfer, for the REVISE Investigators and the Canadian Critical Care Trials Group

**Affiliations:** a McMaster University, Hamilton, Canada; b University of Melbourne, Melbourne, Australia; c Université Laval, Québec City, Canada; d University of Toronto, Toronto, Canada; e King Abdullah International Medical Research Center and King Saud Bin Abdulaziz University for Health Sciences, Riyadh, Saudi Arabia; f University of Alberta, Edmonton, Canada; g King's College, London, United Kingdom; h Al-Amiri Hospital, Kuwait City, Kuwait; i The George Institute, Sydney, Australia

**Keywords:** Adjudication, Clinical trials, Gastrointestinal bleeding, Critical care

## Abstract

**Background:**

Ascertainment of the severity of the primary outcome of upper gastrointestinal (GI) bleeding is integral to stress ulcer prophylaxis trials. This protocol outlines the adjudication process for GI bleeding events in an international trial comparing pantoprazole to placebo in critically ill patients (REVISE: Re-Evaluating the Inhibition of Stress Erosions). The primary objective of the adjudication process is to assess episodes submitted by participating sites to determine which fulfil the definition of the primary efficacy outcome of clinically important upper GI bleeding. Secondary objectives are to categorize the bleeding severity if deemed not clinically important, and adjudicate the bleeding site, timing, investigations, and treatments.

**Methods:**

Research coordinators follow patients daily for any suspected clinically important upper GI bleeding, and submit case report forms, doctors' and nurses’ notes, laboratory, imaging, and procedural reports to the methods center. An international central adjudication committee reflecting diverse specialty backgrounds conducted an initial calibration exercise to delineate the scope of the adjudication process, review components of the definition, and agree on how each criterion will be considered fulfilled. Henceforth, bleeding events will be stratified by study drug, and randomly assigned to adjudicator pairs (blinded to treatment allocation, and study center).

**Results:**

Crude agreement, chance-corrected agreement, or chance-independent agreement if data have a skewed distribution will be calculated.

**Conclusions:**

Focusing on consistency and accuracy, central independent blinded duplicate adjudication of suspected clinically important upper GI bleeding events will determine which events fulfil the definition of the primary efficacy outcome for this stress ulcer prophylaxis trial.

**Registration:**

NCT03374800 (REVISE: Re-Evaluating the Inhibition of Stress Erosions)

## 1 Introduction

Central adjudication can play a key role in many large clinical trials to help ensure a consistent, independent, accurate assessment of several aspects of trial conduct. For example, adjudication may be used to evaluate participant eligibility, assess protocol adherence, examine serious adverse events, or ascertain outcomes.

Outcomes for randomized trials such as mortality are objective and less prone to random and systematic error than morbidity outcomes. For many non-fatal trial endpoints, a central blinded adjudication process can help to establish systematic application of the outcome definition used in a trial. This is particularly important in open-label trials to protect against differential misclassification, as preferred by regulatory authorities such as the European Medicine Agency [1,2] and the United States Food and Drug Administration (FDA) [3]. Beyond standardized definitions [4], independent uniform identification and structured review of major outcomes through central event adjudication is used to enhance the validity of outcome measures in some randomized trials, particularly bleeding and myocardial infarction in cardiovascular trials [5].

In critical care trials of stress ulcer prophylaxis, clear documentation of an upper gastrointestinal (GI) bleeding event is challenging because, even in the context of a specific definition, the interpretation of bleeding severity within and across centers may vary. Further, thresholds for interventions may differ in response to a clinical event, such that it may be difficult to fulfill an outcome definition consistently which incorporate diagnostic tests and treatments across international jurisdictions. Using the definition of clinically important upper GI bleeding as a trial endpoint may benefit from methodology to ensure that outcomes are captured and labelled as intended [Table 1]. This protocol describes the methods of the central adjudication process for upper GI bleeding events in the REVISE Trial (Re-Evaluating the Inhibition of Stress Erosions). REVISE is an international randomized, stratified, concealed, blinded, parallel group trial comparing pantoprazole versus placebo in invasively ventilated critically ill adults [6].

**Table 1:** Definition of clinically important upper gastrointestinal bleeding.

The presence of overt GI bleeding, defined as one of the following:
• Hematemesis • Overt oro/nasogastric bleeding (frank blood or coffee-ground oro/nasogastric aspirate) • Melena • Hematochezia

Plus one of the following in the absence of other causes:
• hemodynamic change defined as a spontaneous decrease in mean arterial pressure or non-invasive systolic or diastolic blood pressure of ≥20 mmHg, or an orthostatic increase in pulse rate of ≥20 beats/minute and a decrease in systolic blood pressure of ≥10 mmHg, with or without vasopressor initiation or increase • vasopressor initiation • hemoglobin decrease of ≥2 g/dl (20 g/L) within 24 h of bleeding • transfusion of ≥2 units packed red blood cells within 24 h of bleeding • therapeutic intervention (e.g., therapeutic endoscopy, angioembolization, surgery)

Legend for Table 1: In this table we define the primary efficacy outcome for the REVISE Trial, which is adjudicated as clinically important upper gastrointestinal bleeding occurring in the ICU or resulting in ICU readmission during the index hospital stay, up to 90-days post-randomization. Criteria comprising the definition are shown here.

The **overall goal** of central adjudication of bleeding events is to minimize random and systematic error that may affect the primary efficacy outcome of clinically important upper GI bleeding. **The primary objective** of the adjudication process is to assess all episodes identified by site investigators as a suspected upper GI bleeding event and to determine which events fulfil the trial definition of the primary efficacy outcome of clinically important upper GI bleeding. The **secondary objectives** are to categorize the severity of the GI bleed if deemed not clinically important and adjudicate the bleeding site, timing, investigations and treatments.

## 2 Methods

### 2.1 Source data

Local research coordinators in each participating REVISE center prospectively follow enrolled patients and complete a GI Bleeding Outcome case report form for each suspected upper GI bleeding event [7]. Research coordinators collect and de-identify doctors' and nurses' notes, as well as reports of relevant tests and treatments from patients’ medical chart (whether an electronic medical record, paper-based, or hybrid medical record). Local site investigators at each participating hospital review and affirm the foregoing data with the research coordinator and approve submission of the GI bleeding outcome case report form. This information is submitted to the REVISE methods center where it undergoes validation in 3 levels, assessing missing data, internal consistency across items, and generating queries to the participating center as needed, followed by an overall review of the patient trajectory, and chart closure when all data are complete and confirmed.

### 2.2 Adjudication packages

For each patient, the methods center will prepare adjudication packages that will be held centrally on a cloud-based server and accessed by adjudicators using password-protected authentication. Adjudicators will not access the entire trial database – just the patients they are adjudicating. Packages will include the following de-identified source data:

a) The GI bleeding outcome case report form (including the center's documentation of the presentation, severity, and start date of the bleed, as well as tests and treatments received); b) Doctors' and nurses' notes for the days of the bleeding event; c) Reports of relevant diagnostic tests (e.g., laboratory data, diagnostic endoscopy, imaging); d) Reports of relevant treatments (e.g., transfusions, therapeutic endoscopy, embolization, surgery); e) All other case reports forms except for those related to allocation, dose dispensing, and other outcomes (e.g., screening, baseline, daily data, final status); and f) The GI bleeding adjudication form (formatted with closed response options for binary and categorical data, and open response options for notes).

### 2.3 Adjudication process

All central adjudication will be performed blinded to a) study drug, b) participating center, and c) assessments of other adjudicators. The main role of the adjudicator is to judge the bleeding episodes and determine whether each event fulfills the definition of clinically important upper GI bleeding, which is the primary outcome of the trial. Completion of other aspects of the adjudication form will assist the adjudicator in making this assessment and serves to address the secondary objectives.

If during the adjudication process, additional questions arise about data submitted from a participating center, the methods center will contact the relevant research coordinator and site investigator to clarify information. If necessary to help ensure uniform application of the GI bleeding definition based on accurate locally submitted information, this liaison with participating centers will serve as an additional step in source data verification.

### 2.4 Adjudication committee

The GI bleeding adjudication committee is comprised of intensivists with diverse backgrounds in internal medicine, gastroenterology, nephrology, surgery, trauma and anesthesiology, representing participating centers in Canada, Australia, Saudi Arabia, Kuwait and the United Kingdom, all of whom have specialty training in critical care. Committee members will independently assess each event using a GI bleeding adjudication form in English, pre-tested by two investigators prior to the calibration exercise.

Material in the adjudication packages will be translated from French or Portuguese into English as necessary. For any bleeding events in Brazil, the medical and nursing notes and relevant hospital reports will be translated from Portuguese into English by the lead Trial Coordinator at the Brazilian methods center who is fluently bilingual; these notes will then be checked by the lead physician investigator. Medical charting in Canada is completed in French in some centers in the province of Quebec; for these patients, the medical and nursing notes and relevant hospital reports will be translated from French into English by the lead physician investigator at the Quebec methods center who is fluently bilingual. If there are any particularly challenging passages that either of the pair of adjudicators questions, a second translation will be requested, and this will be noted in our audit trail.

### 2.5 Calibration exercise

A calibration exercise preceded the adjudication process [8]. **The objectives of the calibration exercise** were to delineate the scope of the adjudication process, review components of the definition of clinically important upper GI bleeding and agree on how each criterion will be adjudicated as fulfilled.

### 2.6 Calibration preparation

Email and telephone discussions were held by investigators prior to 3 calibration meetings to refine an initial adjudication case report form that adjudicators would complete for all suspected GI bleeding episodes and to develop of a standard operating procedure.

**The first meeting** involved committee review of 10 suspected GI bleeding events reported by sites to the methods center. Following their review of the adjudication package and their independent completion of the initial adjudication case report form, 10 committee members met by videoconference. Primed by their independent adjudication of these 10 bleeding events, committee members identified areas of concordance and discordance. Each committee member discussed their rationale for judging bleeding severity, and how they interpreted clinical information in the packages, such as physiologic changes comprising the definition which are judged to be ‘in the absence of other causes’.

*Decisions about the adjudication process* were: a) If a center reported overt bleeding (hematemesis, bloody nasogastric aspirate, hematochezia or melena), but no other clinically important upper GI bleeding criteria were fulfilled, the committee will adjudicate the event as a minor GI bleeding; b) The committee will not adjudicate whether the bleeding event could have been prevented by acid suppression.

*Decisions about the GI bleeding adjudication form* were: a) One question was deleted (i.e., ‘did this start as a minor bleed?’); b) The methods center will henceforth pre-populate with the patient ID, initials, the bleeding start date and study day and end data (if known), and adjudicator identification number; c) The form will be created as a fillable PDF.

No data were analyzed for agreement statistics at this stage.

**The second meeting** followed review of a second set of 10 suspected GI bleeding events. The committee met by videoconference. to discuss each suspected event in detail while initially blinded to each other's assessment. After ratifying the criteria comprising the definition, the committee focus was on whether each event fulfilled the definition of clinically important upper GI bleeding. Discussion ensued to identify areas of disagreement and further calibrate among committee members.

*Decisions about the adjudication process* were: a) If a patient has more than one bleeding event, each will be adjudicated individually in that patient's package (acknowledging that the events may be adjudicated as non-independent distinct bleeding events, or as one intermittent bleeding event); b) Although rare, a patient could be adjudicated to have both upper and lower GI bleeding events on the same study day.

*Decisions about the GI bleeding adjudication form* were: a) Variables were reordered to more clearly distinguish the primary efficacy outcome of clinically important GI bleeding from other degrees of severity, and other sites of GI bleeding; b) A phrase indicating that a minor bleed could be associated with transfusion was deleted to retain distinction between minor bleeding and the definition of clinically important upper GI bleeding that may be fulfilled by transfusion of 2 units of packed red blood cells.

Crude agreement was 80% on the primary trial outcome at this stage.

**The third meeting** followed committee re-adjudication of the first set of 10 GI bleeding events which had originally been adjudicated to prime discussion for the first meeting, but which had not been reviewed in detail. Considering discussion and decisions to this point, members had the opportunity to modify their adjudication forms prior to the meeting. Adjudicators met by videoconference to discuss each patient in turn.

*Decisions about the adjudication process* were: a) The presence of only occult, microscopic or guaiac positive test results of either gastric secretions or stool will be insufficient alone to constitute minor GI bleeding; b) No further changes were made about the adjudication process for the primary trial outcome.

**Fig. 1:**
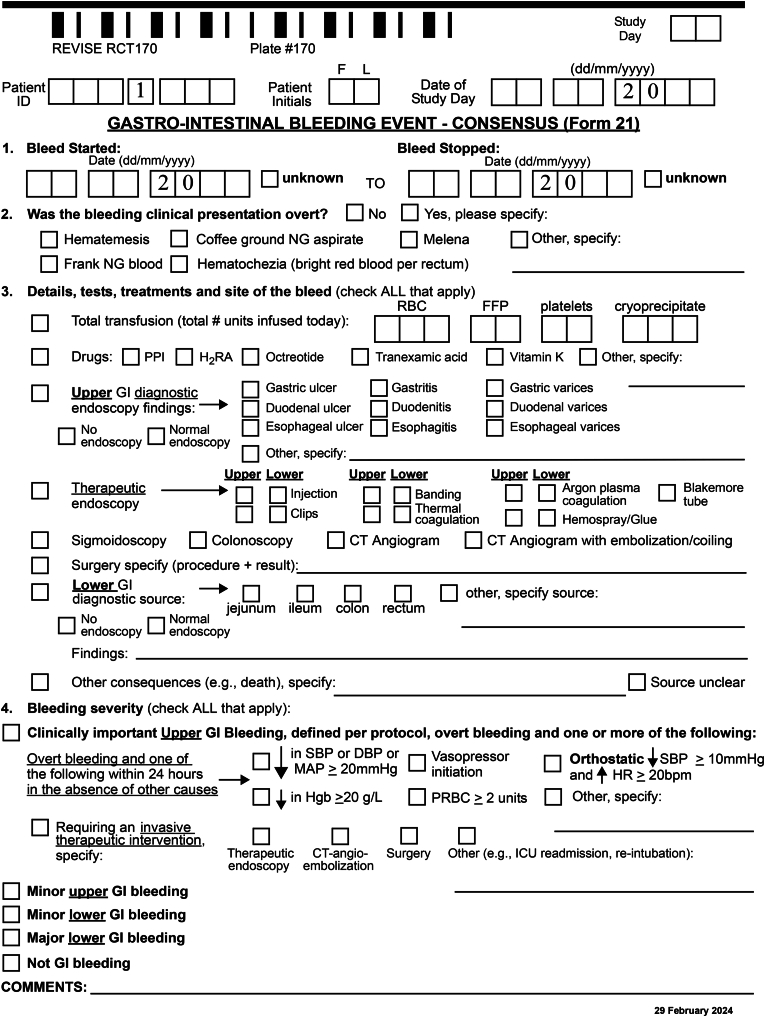
Gastrointestinal bleeding adjudication central adjudication process.

*Decisions about the bleeding adjudication form:* None. The final adjudication case report form is shown in Fig. 1.

Crude agreement was 100% on the primary trial outcome at this stage.

### 2.7 Main adjudication process: random allocation of events

After the calibration exercise among adjudication committee members and consensus on the first 20 cases, duplicate event adjudication will ensue. Each suspected GI bleeding event will be reviewed by 2 adjudicators (one adjudicator who will review all patients, and one adjudicator who is randomly assigned from the committee). A database manager not involved in REVISE will randomize bleeding events stratified by study drug (pantoprazole versus placebo).

### 2.8 Adjudicator agreement

**Fig. 2:**
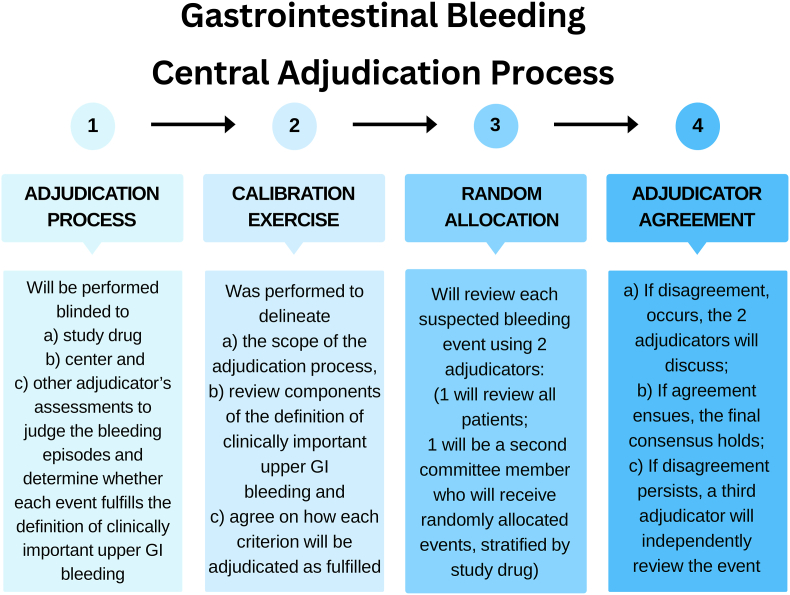
Gastrointestinal bleeding event adjudication consensus form.

In the case of disagreement, the 2 adjudicators will meet by videoconference to discuss. If agreement ensues, the final consensus classification holds. If upon discussion, the 2 adjudicators remain in disagreement, a third adjudicator who also participated in the calibration exercise will independently review the event blinded to the prior adjudication results. The third adjudicator's assessment will hold. If the event that the third adjudicator does not agree with either of the prior 2 adjudicators, the final assessment will be made by the third adjudicator following discussion amongst the 3 adjudicators. This process is depicted in Fig. 2.

## 3 Analysis

For the results section of the main manuscript of the REVISE trial, events adjudicated as clinically important upper GI bleeding episodes will be reported*.* The main manuscript will report the hazard ratio and 95% confidence interval for the primary efficacy outcome of clinically important upper GI bleeding comparing patients receiving pantoprazole versus placebo as adjudicated. For suspected clinically important upper GI bleeding, we will report the overall adjudicator concordance by crude agreement and by chance-corrected agreement using kappa [9] or chance-independent agreement using phi [10] if data have a skewed distribution, based on 2 adjudicators per event.

In the planned subsequent publication, we will assess whether the adjudication influenced the trial outcome, we will report the same metric using events as reported by participating sites and discuss similarities or differences in conclusions drawn from using adjudicated events versus using unadjudicated events. We will not perform a statistical test comparing these metrics, as the bleeding events will be non-independent, occurring in the same patients. All analyses will be performed using SAS version 9.4.

## 4 Results

For this stress ulcer prophylaxis trial, upper GI bleeding severity will be established by physiologic parameters, laboratory values, diagnostic investigations and therapeutic interventions according to the pre-specified definition of clinically important upper GI bleeding. Following the calibration exercise, to help ensure consistent interpretation of the definition and minimize confounding, we will centrally adjudicate all suspected clinically important upper GI bleeding events submitted to the Methods Center. These will be stratified by treatment allocation and randomly assigned to pairs of adjudicators blinded to treatment allocation. If needed, upon review of the first 10 cases by each pair, re-calibration will occur.

## 5 Discussion

Few morbidity endpoints are completely invulnerable to random and systematic error; misclassification of even a small number of events may bias trial results, especially when outcomes are uncommon. For morbidity endpoints whose definitions have components that may benefit from more uniform interpretation across centers, trialists face several decisions about adjudication methods [11]. These include the need to address the size and composition of the committee, training requirements, whether to use adjudication teams, what outcomes will be adjudicated, how cases will be allocated to adjudicators, to what extent the review will be blinded and independent, how the magnitude of agreement will be reported, how consensus will be achieved, whether feedback will be given throughout the process, and whether study results vary with and without adjudication.

Outcome misclassification can lead to erroneous estimates of treatment effect; therefore, strategies to reduce misclassification are integral to clinical trials. However, few studies have been published about whether, when, which and how trial outcomes can be enhanced by central adjudication [12]. A systematic review of different approaches to adjudication showed that they can impact on trial results, depending on local misclassification rates [13], and outcomes in unblinded trials may be particularly vulnerable. For outcomes that do not require much experience or specific training, site determination and central adjudication may generate comparable results. While adjudication has not been definitively shown to improve the ability to determine treatment effects, if rigorously conducted, it can increase confidence in trial results, particularly those based on physiologic changes, which may prompt tests and treatments that are incorporated into definitions. Adjudication strategies are not always reported in trials using such methods, as research is sparse on this topic, and yet is particularly relevant for an outcome with an anticipated low event rate. This study will generate evidence to inform methodologic approaches about adjudication in diverse fields outside of critical care.

Strengths of this protocol include *a priori* reporting of methods to centrally adjudicate the primary efficacy outcome for an international stress ulcer prophylaxis trial. Data contributing to the primary outcome are readily collected by participating sites, validated at the methods center, monitored or audited by the methods center, and adjudicated. Rather than engaging a large number of site assessors, we used a small number of central adjudicators representative of the diversity of the recruiting centers, thus helping to generate, discuss and refine the process of adjudication [13,14]. Our methods accord with recommendations regarding speciality-qualified, task-trained assessors, and adjudication of all suspected events as submitted by participating centers [5], consistent with a low false-negative rate for this uncommon outcome. The adjudication methodology will include stratified randomization of charts to adjudicators, as well as adjudicator blinding to center, study drug and each other's assessments [15], using an efficient web-based system. Each suspected episode will be adjudicated to ascertain whether a bleed fulfils the definition of clinically important upper GI bleeding, and provide another layer of source verification, if needed. This process will also address the secondary objectives of severity (if not clinically important), site (if known), presentation, timing and consequences (tests, treatments and vital status). The analysis will include crude, chance-corrected, and potentially chance-independent agreement metrics. We will examine the effect of adjudication on the trial primary outcome and report the findings.

Limitations of this approach include the reality that GI bleeding severity can be viewed on a continuum, whereas this trial endpoint is designed as binary. While clinically important upper GI bleeding requires judgement when interpreting alignment with the definition, the propensity to fulfil the definition may vary across centers, depending on individual clinician comfort, local practice patterns, transfusion policies and procedural accessibility. However, across sites, patient-specific factors and laboratory values at the time of a bleed may also influence whether a definition is fulfilled. For example, a patient with septic shock who has upper GI bleeding may be more likely to receive vasopressors than a patient without shock. A hemoglobin decrease from 75 g/dl to 55 g/dl would be more likely to prompt transfusion than a hemoglobin decrease from 110 g/dl to 90 g/dl, *all else being equal*. However, such clinical situations would likely be similarly distributed in the 2 arms of a large randomized blinded trial.

As identified by literature reviews, *between-study* differences in definitions for the same nominal endpoint of clinically important upper GI bleeding may affect pooled estimates of bleeding rates, the strength of bleeding risk factors, and effect sizes for prevention strategies. The perspective for this protocol is reproducibility *within-study*, to apply the definition of clinically important upper GI bleeding as consistently as possible in this trial, as reliance on clinical databases is insufficient for this purpose. Leveraging electronic medical records or artificial intelligence to mine or model morbidity endpoints is an alternative approach used in registry-based randomized trials when precise ascertainment is less relevant or considered sufficiently reliable [5].

In summary, to accurately capture the number and affirm the severity of bleeding episodes in REVISE, every potentially clinically important upper GI bleeding event reported by a participating center will be independently adjudicated in duplicate by a calibrated central adjudication committee. Given that there is inherent uncertainty in the adjudication process even with standardized criteria, we convened an international group of experienced critical care practitioners and investigators for this process and conducted a calibration and task-specific training exercise. This approach will strengthen the inferences about the treatment effect of pantoprazole versus placebo among invasively ventilated patients by helping to uniformly apply the criteria for the primary efficacy outcome across centers, among adjudicators, between adjudicator pairs, across centers, and over the duration of the trial.

## Bleeding adjudication committee members

Drs. Abdulrahman Al-Fares, Yaseen Arabi, Deborah Cook, Adam Deane, Joanna C. Dionne, Simon Finfer, François Lauzier, John C. Marshall, Marlies Ostermann, M. Elizabeth Wilcox. The methods center project lead is Nicole Zytaruk. The analyst is Diane Heels-Ansdell.

## Contributorship statement

**Concept and design:** Deborah Cook, Adam Deane, Joanna C. Dionne, François Lauzier, John C. Marshall, Yaseen Arabi, M. Elizabeth Wilcox, Marlies Ostermann, Abdulrahman Al-Fares, Diane Heels-Ansdell, Nicole Zytaruk, Simon Finfer.

**Acquisition, analysis, or interpretation of data:** Deborah Cook, Adam Deane, Joanna C. Dionne, François Lauzier, John C. Marshall, Yaseen Arabi, M. Elizabeth Wilcox, Marlies Ostermann, Abdulrahman Al-Fares, Diane Heels-Ansdell, Nicole Zytaruk, Simon Finfer.

**Drafting of the manuscript:** Deborah Cook, Adam Deane, Joanna C. Dionne, François Lauzier, John C. Marshall, Yaseen Arabi, M. Elizabeth Wilcox, Marlies Ostermann, Abdulrahman Al-Fares, Diane Heels-Ansdell, Nicole Zytaruk, Simon Finfer.

**Critical revision of the manuscript for important intellectual content:** Deborah Cook, Adam Deane, Joanna C. Dionne, François Lauzier, John C. Marshall, Yaseen Arabi, M. Elizabeth Wilcox, Marlies Ostermann, Abdulrahman Al-Fares, Diane Heels-Ansdell, Nicole Zytaruk, Lehana Thabane, Simon Finfer.

**Statistical analysis:** Nicole Zytaruk, Diane Heels-Ansdell, Lehana Thabane.

**Obtainedfunding:** Deborah Cook, Adam Deane, Joanna C. Dionne, François Lauzier, John C. Marshall, Yaseen Arabi, M. Elizabeth Wilcox, Marlies Ostermann, Diane Heels-Ansdell, Nicole Zytaruk, Lehana Thabane, Simon Finfer.

**Administrative, technical, or materialsupport:** Diane Heels-Ansdell, Nicole Zytaruk.

**Data Integrity:** Deborah Cook, Adam Deane, Joanna C. Dionne, François Lauzier, John C. Marshall, Yaseen Arabi, M. Elizabeth Wilcox, Marlies Ostermann, Abdulrahman Al-Fares, Diane Heels-Ansdell, Nicole Zytaruk, Simon Finfer.

## Funding statement

REVISE is funded by peer-reviewed grants [10.13039/501100000024Canadian Institutes of Health Research 201610PJT-378226-PJT-CEBA-18373, 10.13039/501100000024Canadian Institutes of Health Research 202207CL3-492565-CTP-CEBA-19215], and the 10.13039/501100000024Canadian Institutes for Health Research Accelerating Clinical Trials Fund [ACT Consortium RFA-1 Application], as well as the 10.13039/100010182Hamilton Academy of Health Sciences Organization [HAH-22-009], and funds from St. Joseph's Healthcare Hamilton and McMaster University. The National Health and Medical Research Council of Australia grant [GNT1124675] funds enrolment in Australia. REVISE was approved by the 10.13039/501100000272National Institute for Health Research (NIHR) in the UK as a Portfolio Study [CPMS ID 45782], eligible for support from the 10.13039/501100000272NIHR Clinical Research Network. [https://www.nihr.ac.uk/researchers/collaborations-services-and-support-for-your-research/run-your-study/crn-portfolio.htm]. This trial received no support from the commercial or private sector. The funders/sponsors have no role in the conception, design, conduct, oversight, analysis, interpretation, write-up, review or approval of the manuscript, or decision to submit the manuscript for publication.

## Career award funding

Dr. D Cook holds a Research Chair in Knowledge Translation in Critical Care from the Canadian Institutes for Health Research. Dr. J Dionne holds an Early-Career Investigator Award from McMaster University's Department of Medicine. Dr. F Lauzier is a recipient of a Research Career Award from the *Fonds de la recherche du Québec-Santé*. Dr. J Marshall holds the Unity Health Chair in Trauma Research. Dr S Finfer holds a Leadership Fellowship from the National Health and Medical Research Council of Australia.

## Data sharing statement

Following the publication of REVISE, the dataset will be used for secondary observational studies addressing additional hypothesis-driven questions (e.g., predictors of gastrointestinal bleeding). Access by REVISE investigators will follow a submitted rationale, analysis plan and approval by the Management Committee. Requests for access to the dataset by external investigators will be considered following a submitted rationale, analysis plan and approval by the Management Committee and research ethics boards as relevant. Requirements will be stipulated in a pre-specified data sharing agreement. Only de-identified data will be provided and will be transferred via a secure web portal.

## Ethics approval

The Hamilton Integrated Research Ethics Board is the Ethics Board of Record (CTO Project ID: 1360). Relevant Research Ethics Boards (REBs) and/or Human Research Ethics Committees (HRECs) of each participating hospital and/or region approved REVISE. These include but are not limited to: Australia: Northern Sydney Local Health District Human Research Ethics Committee and Mater Misericordiae Ltd Human Research Ethics Committee; Brazil: Comissão Nacional de Ética em Pesquisa; Canada: Hamilton Integrated Research Ethics Board; Kuwait: Ministry of Health Standing Committee for Coordination of Health and Medical Research; Pakistan: Maroof Institutional Review Board; Saudi Arabia: Ministry of National Guard Health Affairs Institutional Review Board: United Kingdom: Hampshire B Research Ethics Committee; United States: Institutional Review Board of the Nebraska Medical Center.

## CRediT authorship contribution statement

**Deborah Cook:** Conceptualization, Data curation, Funding acquisition, Investigation, Methodology, Resources, Validation, Writing – original draft, Writing – review & editing. **Adam Deane:** Conceptualization, Data curation, Funding acquisition, Investigation, Methodology, Validation, Writing – original draft, Writing – review & editing. **Joanna C. Dionne:** Conceptualization, Data curation, Funding acquisition, Investigation, Methodology, Validation, Writing – review & editing, For the REVISE Investigators and the Canadian Critical Care Trials Group, Funding acquisition, Investigation, Writing – review & editing. **François Lauzier:** Conceptualization, Data curation, Funding acquisition, Investigation, Methodology, Writing – original draft, Writing – review & editing, Validation. **John C. Marshall:** Conceptualization, Data curation, Funding acquisition, Investigation, Methodology, Writing – review & editing, Validation, Writing – original draft. **Yaseen M. Arabi:** Data curation, Funding acquisition, Investigation, Methodology, Writing – original draft, Writing – review & editing, Validation. **M. Elizabeth Wilcox:** Data curation, Funding acquisition, Investigation, Methodology, Writing – review & editing. **Marlies Ostermann:** Data curation, Funding acquisition, Investigation, Methodology, Validation, Writing – review & editing. **Abdulrahman Al-Fares:** Data curation, Investigation, Methodology, Validation, Writing – review & editing. **Diane Heels-Ansdell:** Data curation, Formal analysis, Funding acquisition, Investigation, Methodology, Project administration, Writing – review & editing, Conceptualization, Resources, Software. **Nicole Zytaruk:** Conceptualization, Data curation, Funding acquisition, Investigation, Methodology, Project administration, Software, Writing – original draft, Writing – review & editing, Resources, Supervision. **Lehana Thabane:** Formal analysis, Funding acquisition, Investigation, Methodology, Supervision, Writing – review & editing. **Simon Finfer:** Conceptualization, Data curation, Funding acquisition, Investigation, Methodology, Supervision, Writing – original draft, Writing – review & editing.

## Declaration of competing interest

The authors declare that they have no known competing financial interests or personal relationships that could have appeared to influence the work reported in this paper.

## Data Availability

The authors do not have permission to share data.
